# Genetic determinism of cortisol levels in pig

**DOI:** 10.3389/fgene.2025.1461385

**Published:** 2025-03-12

**Authors:** Elena Terenina, Nathalie Iannuccelli, Yvon Billon, Katia Fève, Laure Gress, Darya Bazovkina, Pierre Mormede, Catherine Larzul

**Affiliations:** ^1^ GenPhySE, INRAE, ENVT, Université de Toulouse, Castanet Tolosan, France; ^2^ GenESI, INRAE, Surgères, France; ^3^ Federal Research Center Institute of Cytology and Genetics, Siberian Division of the Russian Academy of Science, Novosibirsk, Russia

**Keywords:** pig, HPA axis, glucocorticoid receptor, CBG, adrenocorticotropic hormone, robustness

## Abstract

In facing the challenge of sustainability, animal breeding provides the option to improve animal robustness. In the search for new selection criteria related to robustness, the hypothalamic–pituitary–adrenocortical (HPA) axis is studied as a major neuroendocrine system involved in metabolic regulations and adaptive responses. Indeed, HPA axis activity is strongly influenced by genetic factors acting at several levels of the axis. The adrenocorticotropic hormone (ACTH) stimulation test has long been used to analyze interindividual and genetic differences in HPA axis activity in several species, including pigs. To uncover the genetic determinism of HPA activity and its influence on functional traits and robustness, a divergent selection experiment was carried out for three generations in a Large White pig population based on plasma cortisol levels measured one hour after injection of ACTH. In the present study the response to selection was very strong (confirming our previous studies), with a heritability value of cortisol level after ACTH injections reaching 0.64 (±0.03). The difference between the two divergent lines was around five genetic standard deviations after three selection steps. A genome-wide association study pointed out the importance of the glucocorticoid receptor gene (*NR3C1*) in this response. The measurement of plasma corticosteroid-binding globulin (CBG) binding capacity excluded any significant role of CBG in this selection process. The phenotypic effect of selection on body weight and growth rate was modest and/or inconsistent across generations. The HPA axis, a major neuroendocrine system involved in adaptation processes is highly heritable and responsive to genetic selection. The present experiment confirms the importance of glucocorticoid receptor polymorphism in genetic variation of HPA axis activity–in addition to the previously demonstrated role of CBG gene polymorphism. Further studies will explore the effect of this divergent selection on production and robustness.

## 1 Introduction

Animal production is increasingly challenged by antibiotics use, environment control in the context of climate change, feed-food competition and animal welfare. To face these challenges, animal breeding and genetics can provide a sustainable option by improving animal robustness, subject to our ability to provide new selection criteria related to robustness (Rauw and Gomez Raya, 2015). For decades, genetic selection in the pig industry has focused primarily on production traits, which has had negative impacts on health and welfare (as reviewed in Prunier et al., 2010). To promote the sustainability of farming, including animal welfare, it is necessary to reconsider the selection criteria. Introducing new genetic selection factors related to pig health could help reduce the use of antibiotics, which is crucial for addressing public health concerns. Specifically, selecting for robust pigs could enhance their resistance to pathogens. A positive link between hypothalamic-pituitary-adrenocortical (HPA) axis activity and robustness has been previously reported (Mormede et al., 2011; Mormede and Mormede, 2024). The HPA axis plays a crucial role in maintaining physiological balance, including metabolism, brain function, behavior, immune responses, and inflammation. Along with the autonomic nervous system, it also participates in stress and adaptive responses to environmental challenges (see Mormede and Mormede, 2024, for review). The HPA axis activity being strongly influenced by genetic factors (Mormede et al., 2011; Mormede and Mormede, 2024), animal breeding provides an option to improve animal robustness (Mormede and Terenina, 2012). Cortisol, the primary terminal effector of the HPA axis in both humans and pigs, is secreted by the adrenal cortex. This secretion is controlled by the release of corticotropin-releasing factor and vasopressin from the hypothalamus, which in turn stimulate the secretion of adrenocorticotropic hormone (ACTH) from the anterior pituitary gland. Adrenal gland sensitivity to ACTH varies significantly among individuals and was shown to be a highly heritable trait (Mormede and Mormede, 2024).

In pigs, for example, the sensitivity of the adrenal cortex to adrenocorticotropic hormone and the level of circulating corticosteroid-binding globulin (CBG), which transports cortisol in blood, are key mechanisms responsible for genetic differences in circulating cortisol levels (Désautés et al., 2002; Larzul et al., 2015). In a previous study, Hazard et al. (2008) explored the molecular mechanisms of genetic variation in the adrenal gland response to ACTH at the gene expression level. However, there is limited understanding of individual differences in the biological activity of cortisol, the primary glucocorticoid hormone, and the genetic mechanisms involved. Corticosteroid hormones exert their effects through two intracellular receptors (glucocorticoid (GR) and mineralocorticoid receptors), which, upon activation by their ligands, influence the expression of a wide range of genes in various cell types (Necela and Cidlowski, 2004). In pigs, polymorphisms of the GR gene (*NR3C1*) have been identified, along with their functional consequences (Murani et al., 2012; Terenina et al., 2013; Reyer et al., 2016). The ACTH stimulation test has long been used to analyze inter-individual differences and genetic influences on HPA axis activity in several species, including pigs (Hennessy and Jackson, 1987; Hennessy et al., 1988; Von Borell and Ladewig, 1992; Désautés et al., 1999). To understand the genetic determinism of HPA activity and its influence on functional traits and robustness, a divergent selection experiment was carried out for three generations in a Large White pig population based on plasma cortisol levels measured one hour after injection of ACTH (Larzul et al., 2015). Furthermore, the availability of divergent populations will allow the study of the contribution of the HPA axis to production and adaptation traits (robustness).

## 2 Materials and methods

### 2.1 Animals and housing

The base population was the progeny (F0) of a foundation breeding stock of purebred French Large White pigs consisting of 30 boars, present in French artificial insemination (AI) centers and 30 sows. This F0 population was described in a previous publication (Larzul et al., 2015). In order to maximize the initial genetic diversity of the F0 population, AI boars were chosen as unrelated as possible based on their pedigree information. The relationship coefficients were estimated for French Large White AI boars with available for semen collection using the PEDIG software (Boichard, 2002), and the 30 boars the less related were chosen to inseminate 30 Large White sows present on the experimental farm (GenESI, Le Magneraud, France). From that F0 population and throughout the whole selection experiment, piglets were raised on the same experimental farm. At birth, piglets were preferably kept with their mother, except for large size litters (greater than 12) of which excess piglets were removed and adopted by another dam with a smaller litter size. At weaning, at 4 weeks of age, piglets from two to three different litters were regrouped in post-weaning pens of 24 individuals. When the selected lines were established after the first generation of selection (see section 2.2), piglets from different lines were always housed in distinct post-weaning pens. At 70 days of age, each post-weaning pen was divided in two groups of 12 individuals, which were transferred to growing-finishing pens. After weaning, they received food and water *ad libitum*. Starter diet (18.6% protein and 10.8 MJ/kg net energy (NE) on a dry matter basis) was given during the last lactating week and during the first 2 weeks after weaning. Weaner diet (17.5% protein and 10.0 MJ/kg NE) was given from the second week after weaning up to the end of the post-weaning period. A 16% protein and 9.7 MJ/kg NE was given during the growing-finishing period.

### 2.2 Selection

#### 2.2.1 Selection criterion

Animals were selected on the basis of their cortisol level after ACTH injection performed at 6 weeks of age. For the F0 population, details of the procedure were previously published (Larzul et al., 2015). Experiments were done in the morning (8 h–12 h). Piglets were caught one by one in their pen and put on the back in a havoc adjusted to their size and maintained on their back by light restraint. Immediately after a first blood sampling from the jugular vein, they received an ACTH injection in the neck muscles and were released back to their pen. They were caught once again in the same conditions one hour later for blood sampling. Each procedure did not take more than 30 s after catching the animal in the pen. The delay for blood collection after ACTH injection (1 h) corresponds to the peak of the response (Hennessy et al., 1988). Blood samples were collected in tubes with sodium heparin (Vacutainer^®^, Becton-Dickinson, Le Pont de Claix, France) by direct puncture from the jugular vein. During the first two generations (F0 and G1), piglets received synthetic mammalian ACTH(1–24) (Immediate Synacthen; Novartis, Rueil-Malmaison, France) at the dose of 250 μg/animal. Due to Synacthen shortage, piglets from the following generations (G2 and G3) were injected with synthetic porcine ACTH(1–39) (Pepscan Presto B.V., Lelystad, Netherlands) under the same conditions at the dose of 333 μg/animal (equimolar to Synacthen 250 μg/animal). The dose of ACTH was chosen to be maximally stimulating the adrenal cortex. Blood samples were rapidly centrifuged and plasma was frozen at −80°C (F0) or −20°C (G1 to G3) until assay. For G1 to G3 animals, cortisol level was measured within 24 h after collection in order to rapidly select the future reproducers. For F0 animals, plasma total cortisol was measured using a specific direct radio immunoassay (RIA) (GammaCoatTM Cortisol, DiaSorin, Antony, France). During the following generations, *i.e.*, G1 to G3, plasma cortisol was measured by direct automated immunoassay (AIA-1800, Tosoh Bioscience, San Francisco, CA). The selection criterion was the plasma cortisol level measured 1 h after ACTH injection.

#### 2.2.2 Selection experiment

In the base population of pure Large White pigs 298 intact male and female piglets were phenotyped. From these, 14 males were selected, 7 within the highest cortisol values (H line) and 7 within the lowest cortisol values (L line) as founders of the two divergent lines. The boars were chosen from different litters, in order to limit inbreeding in the following generation. Semen collected from those boars was frozen and used to inseminate 62 unselected Large White sows from the same stock as the base population, equally and randomly distributed to each future line. Four or five sows were inseminated per boar. On the following generations, 8 males and 40 females were selected on extreme cortisol values, within lines. Replacement boars and gilts were chosen among first-parity litter progeny. Boars were selected within sire family, and one boar was replaced by one (or two for G1) of its sons. Females were selected on their post-ACTH cortisol level, irrespective of their dam origin. At first parity, each selected boar inseminated five gilts from the same line, only avoiding full or half-sib mating. For each generation, the 80 selected dams were distributed over 3 successive batches. Numbers of animals measured for the selection criterion, per generation, are reported in Table 1.

**TABLE 1:** Number of animals and mean (standard deviation) post-ACTH cortisol level per line and generation.

	N		Mean (ng/mL)	
Generation	Low line	High line	Low line	High line
F0	298		99 (25)	
G1	201	152	105 (21)	121 (25)
G2	403	357	87 (22)	129 (20)
G3	330	313	73 (17)	158 (35)

#### 2.2.3 Additional animals

From the unselected 62 Large White sows, 30 were inseminated a second time with 30 AI Large White boars; 120 animals (G0), 4 per litter, were raised under the same conditions as the animals from the selection experiment and were also measured for the selection criterion. The complete description of this part of the experiment was reported previously (Sautron et al., 2015). As these animals were raised and measured in similar conditions as the animals from the F0 population and the selection experiment, data collected on these animals were added to the present study to provide a larger dataset for statistical analyses.

A schematic representation of the experimental design is provided in Supplementary Figure S1.

#### 2.2.4 Production traits

All piglets were weighed at birth (BW) and at weaning (WW). The average daily gain was thus estimated for the lactating period (ADGL).

### 2.3 Genotyping

The 62 unselected Large White sows, the 120 G0 piglets, the 14 F0 selected boars, the sows and boars selected as reproducers in the G1 and G2 populations as well as the extremes from the G3 population, 16 boars and 80 sows as if they would have been selected as reproducers, were genotyped. DNA was extracted from the tail, cut shortly after birth. The F0, G0 and G1 animals were genotyped using the PorcineSNP60 Beadchip from Illumina (San Diego, CA, United States). The G2 and G3 animals were genotyped with a Geneseek-Neogen GPPHD 80K SNP chip. Additionally, the 14 selected boars from the F0 population and 47 G0 females, with the highest number of selected offspring, among the 62, were genotyped with Affymetrix Axiom^®^ 650K SNP Array for imputation. The following filters were applied for each array: map information on autosomes (Sscrofa 11.1), call rate higher than 95% and minor allele frequency (MAF) higher than 5%. The imputation from 60K or 80K to 650K was performed using Fimpute v2.2 (Sargolzaei et al., 2014) including pedigree information over 5 generations, and 578,947 SNP were finally used for association study (Larzul et al., 2018).

#### 2.3.1 *NR3C1* genotyping

Genotyping of the c.1829C>T mutation of the glucocorticoid receptor gene *NR3C1* (Murani et al., 2012) was performed by allele-specific PCR amplification using the KASP (Kompetitive Allele-Specific PCR) SNP genotyping system followed by fluorescence detection on a QuantStudio™ 6 of Applied Biosystems (end-point fluorescent PCR read) (Semagn et al., 2013). KASP assays were carried out in 5 µL reactions following the manufacturer recommendations with some adjustment: genotyping was performed with 12.5 ng of DNA, a ratio 1:3 of allele-specific primers (0.08 µM for Allele 2 and 0.24 µM for Allele 1) and 0.4 µM of common primers, using KASP V4.0 2X Master Mix (LGC Biosearch Technologies, Middlesex, UK) for 45 cycles of 1 min at 62°C. The primers were defined as followed: a unique common forward primer (up2) for both alleles and two allele specific reverse primers to differentiate the two SNP (All1 and All2):

Kaspar_NR3c1. up2 CCC​CTT​TTG​TGC​CTC​GCT​TCC.

Kaspar_NR3c1_All1. dn2 CTC​CAC​CCC​AGG​GCG​AAC​g.

Kaspar_NR3c1_All2. dn2 CTC​CAC​CCC​AGG​GCG​AAC​a.

A total of 231 animals, with available DNA material, from the F0 population and all individuals from the G3 low line were genotyped. Animals from the high line, based on information provided by the genotyping of the founders of the line, were all considered as homozygous for the wild allele.

#### 2.3.2 Corticosteroid-binding globulin (CBG) binding capacity assay

CBG, the carrier glycoprotein of cortisol in plasma, is a major source of genetic variability in circulating cortisol levels (Désautés et al., 2002; Guyonnet-Duperat et al., 2006). To explore its possible role in the selection process, CBG binding capacity (Bmax) was measured as described previously (Geverink et al., 2006) in plasma from 6-week old female piglets, unselected (G0, N = 30) and from selected lines at the third generation (G3, N = 16 each line).

### 2.4 Statistical analyses

Data for cortisol concentration and CBG binding capacity were normalized with a log transformation. For cortisol levels, preliminary least-squares analyses were performed using the GLM procedure of SAS to estimate the effect of sex, batch within generation and line x generation combination on each trait. For weights measured at birth and weaning, the litter size (LS) was added as a fixed effect. The levels of the litter size (LS) effect were reduced to 4 to avoid marginal effects (with very few observations) of extreme litter sizes (1: LS < 8; 2: 8–12; 3: 13–17; 4: LS > 17).

For genetic analysis, the random effects of common litter and animal were also included in the model, without the line x generation effect. Genetic parameters were estimated with a two-trait animal model. All the ancestors of the recorded animals up to five generations from the F0 animals were taken into account to build the additive relationship matrix. The estimation of genetic parameters was performed with VCE6 (Neumaier and Groeneveld, 1998). Additive genetic breeding values were estimated with BlupF90 (Misztal et al., 2002) using the genetic parameters previously estimated. The response to selection was estimated by averaging predicted breeding values within line and generation.

Association analyses were performed with the R package GenABEL using the FASTA procedure to take into account polygenic effects and kinship based on identity-by-state function estimated from SNP (Aulchenko et al., 2007). The p-values were corrected for genomic control by dividing the observed test statistic by a calculated genomic inflation factor. Associations that passed the threshold of P < 10^–6^ were considered as significant.

CBG binding capacity was compared between experimental groups by analysis of variance and Pearson’s product moment correlation coefficient was used to test for association between CBG binding capacity and cortisol levels.

## 3 Results and discussion

### 3.1 Genetic parameters and response to selection

The heritability value of plasma cortisol concentration measured 1 h after ACTH injection was 0.64 (±0.03). This value is very similar to the previous value (0.68) estimated in the F0 population (Larzul et al., 2015) and confirms the high heritability of this trait.

**FIGURE 1:**
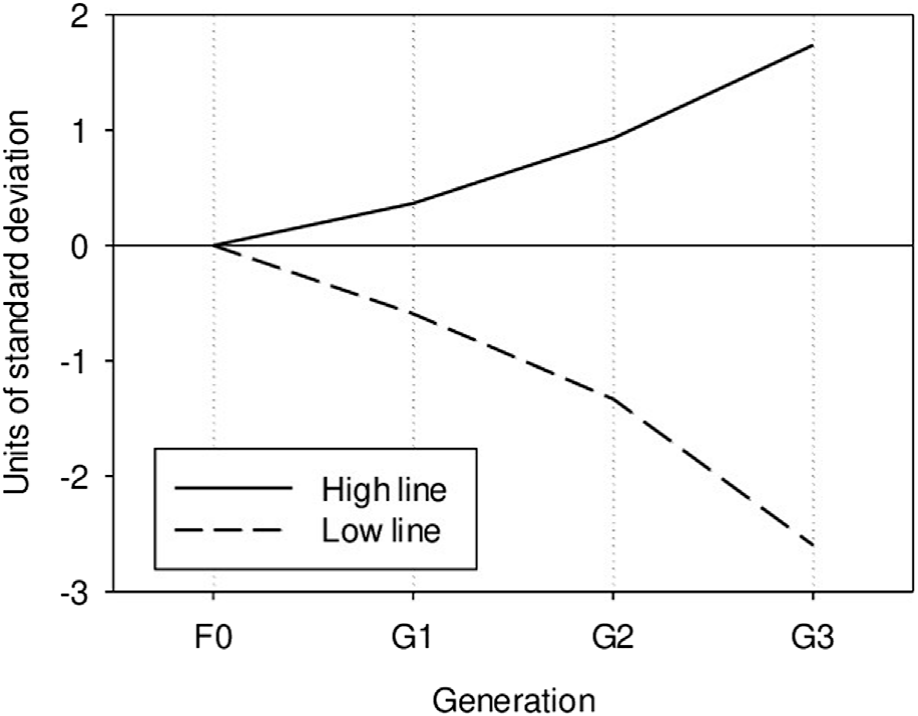
Genetic evolution of post-ACTH cortisol level in the high and low lines, in units of genetic standard deviation.

As expected from the heritability value, the response to selection was large. The difference between the two divergent lines after three selection steps was around five genetic standard deviations (Figure 1). Kadarmideen and Janss (2007) estimated a heritability value of 0.40 for basal cortisol concentration in urine, but the heritability values reached 0.70 when cortisol level was adjusted to creatinine level. Though not measured under the same conditions, these results underline the important influence of the genetic background on cortisol level variability, at rest and under stressful conditions.

**FIGURE 2:**
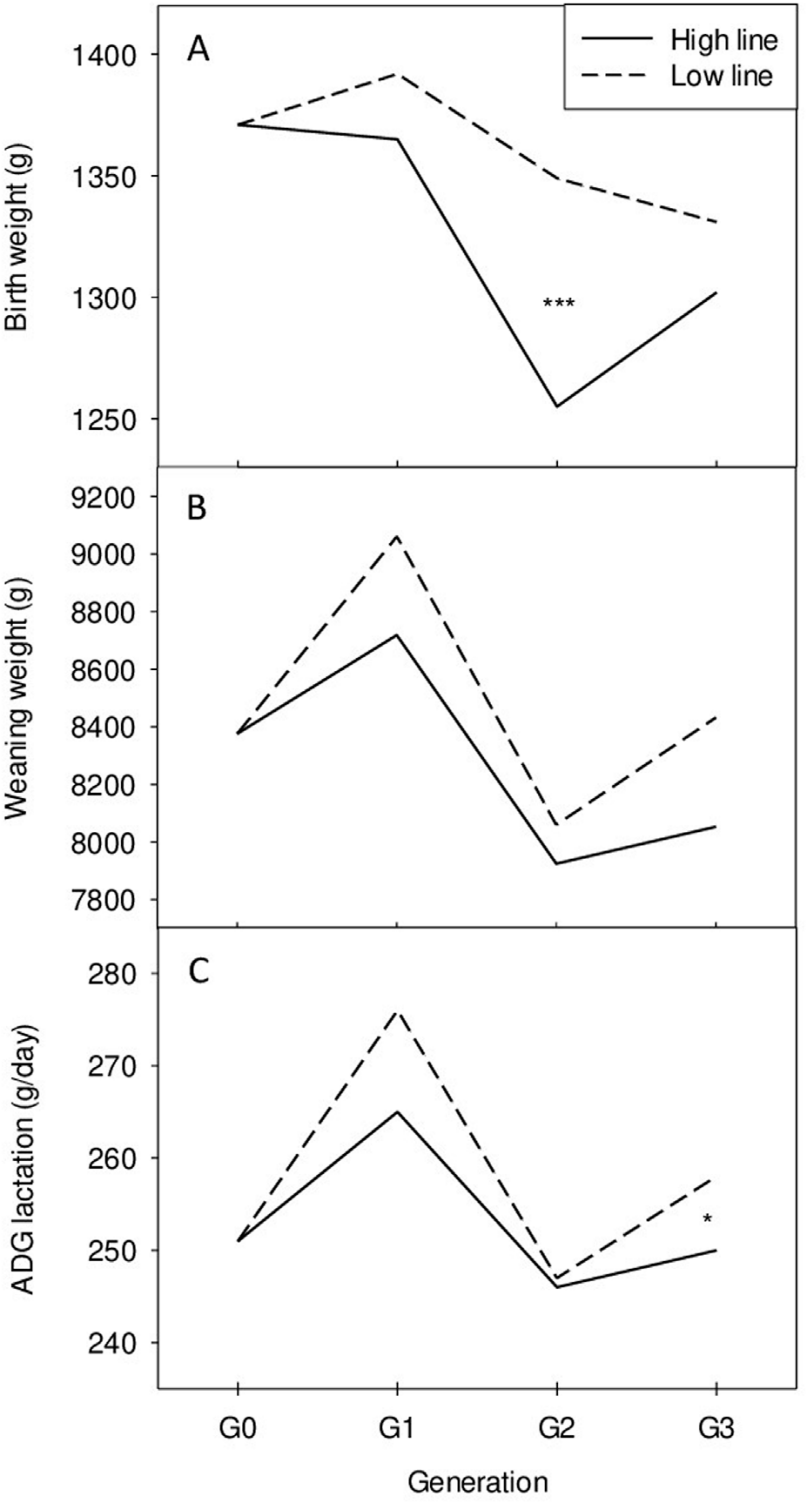
Phenotypic changes across successive generations of selection in the high (H) and low (L) lines. **(A)** Birth weight, **(B)** Weaning weight, **(C)** Average daily gain during lactation. *P < 0.05, ***P < 0.001.

The heritability values for other traits are reported in Table 2 as well as genetic correlations with the selection criterion. All the genetic correlations between cortisol level and weight or growth rate are low. No significant correlated response was thus expected as shown by the absence of marked differences between lines during the whole experiment for these traits as shown in Figure 2 for growth performance until weaning.

**TABLE 2:** Genetic parameters of growth data.

Traits	N	h^2^	se h^2^	r_g_	se r_g_	c^2^	se c^2^
BW	3405	0.18	0.05	−0.04	0.09	0.24	0.02
WW	2491	0.11	0.05	0.00	0.11	0.44	0.03
ADGL	2491	0.07	0.05	0.02	0.13	0.46	0.03

Abbreviations: Birth weight (BW), weaning weight (WW), average daily gain during lactation (ADGL), heritability (h^2^), genetic correlations with cortisol level (r_g_), common litter effect (c^2^) and their standard error (se).

### 3.2 Genome wide association study

**FIGURE 3:**
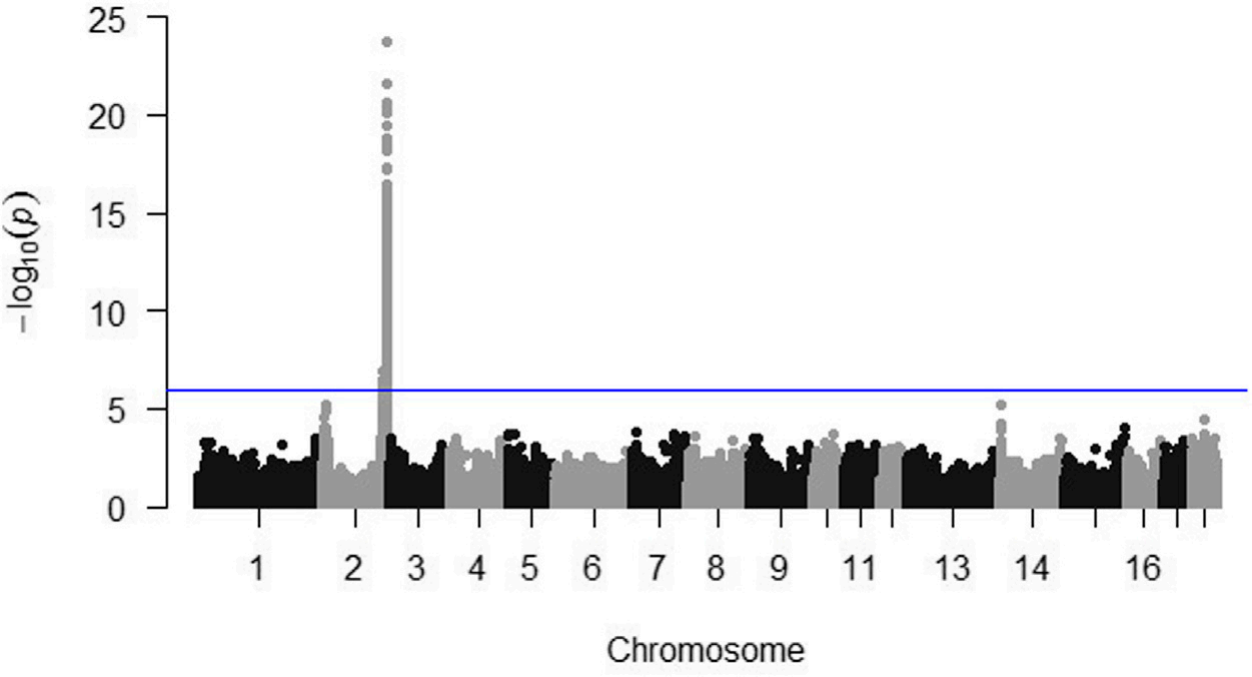
Manhattan plot for post-ACTH cortisol level. The horizontal line indicates the significance threshold (P < 10–6).

A highly significant quantitative trait locus was located on chromosome 2 (Figure 3). The 13 SNP most significantly associated with post-ACTH cortisol level are reported in Table 3 with their chromosomal location and estimate P-value. No SNP was significantly associated with body weights at the P < 10^–6^ value.

**TABLE 3:** The 13 most significant SNPs for post-ACTH cortisol level.

SNP name	Chromosome	Position	Adjusted *P*_value
AX-116716551	2	145045775	5.71E-26
AX-116716547	2	144974676	9.38E-24
AX-116190918	2	145228569	1.10E-22
AX-116190798	2	144792892	2.12E-22
AX-116190803	2	144808932	2.12E-22
AX-116190806	2	144818971	2.12E-22
AX-116190807	2	144822899	2.12E-22
AX-116190809	2	144827094	2.12E-22
AX-116190811	2	144831897	2.12E-22
AX-116190813	2	144836353	2.12E-22
AX-116716529	2	144838566	2.12E-22
AX-116680688	2	144841166	2.12E-22
DRGA0017574	2	144841166	2.12E-22

**FIGURE 4:**
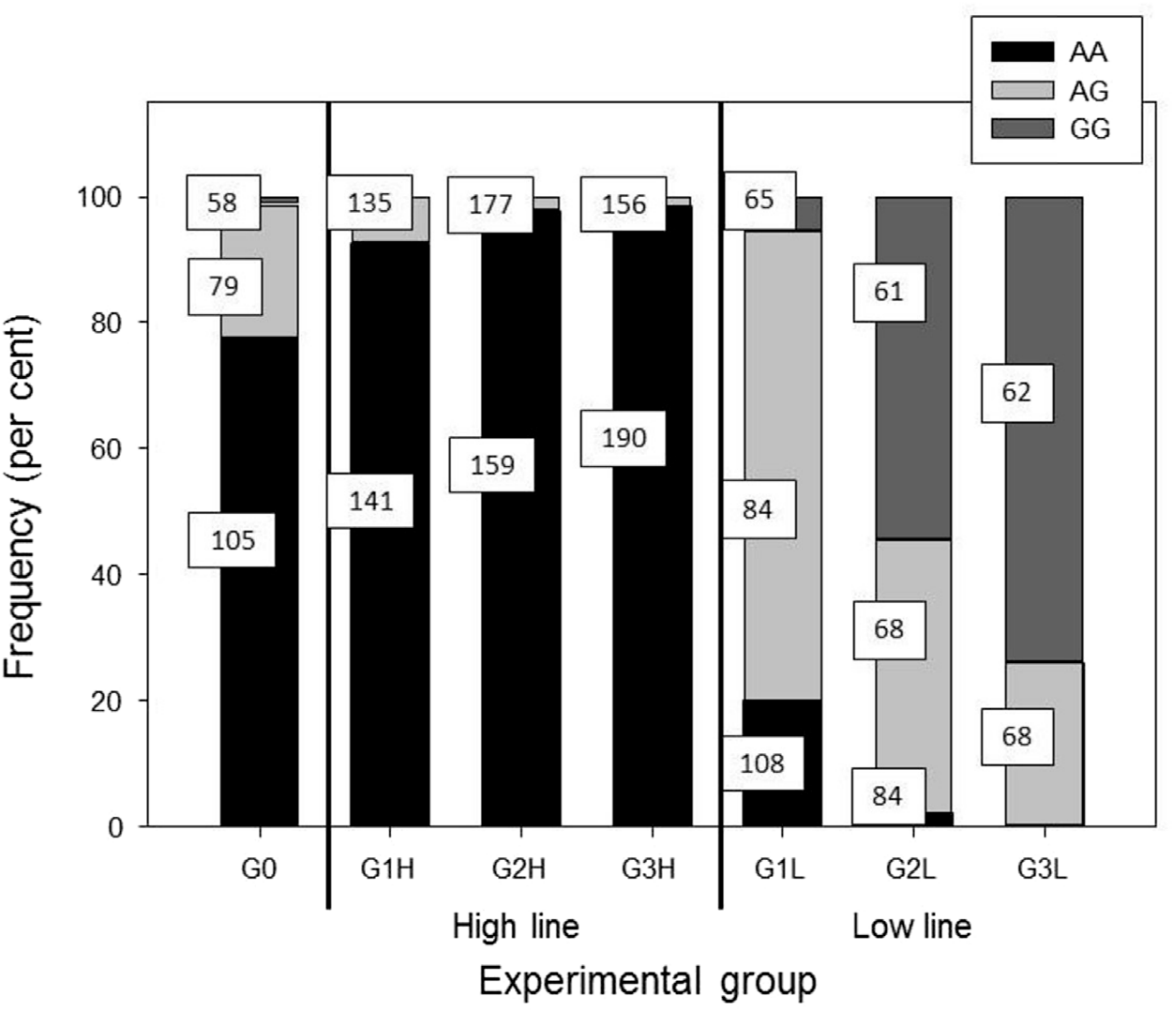
Evolution of the frequency of the genotypes for the SNP DRGA001754. In squares are shown the means of post-ACTH cortisol levels for the respective genotype.

The most significant SNP for post-ACTH cortisol level were located on chromosome 2. One of the SNP most significantly associated with cortisol level, DRGA0017574, was the same as the one reported previously by Murani et al. (2012) with an effect on cortisol level at slaughter and adrenal gland weight. This SNP, as well as the other significant SNP, are located near or within the *NR3C1* gene. Murani et al. (2012) suspected the c.1829C>T substitution to be responsible for hypersensitivity of the glucocorticoid receptor (GR). The mutation was shown to enhance ligand binding and significantly increase GR activation in response to glucocorticoid and non-glucocorticoid steroids (Reyer et al., 2016). Figure 4 shows the evolution of the frequency of the two alleles of the SNP DRGA001754 in both lines during the selection process. It is obvious that the G allele was preferentially selected in the low line, with a large decrease of the AA frequency and an increase in the AG and GG frequencies. Considering that the G allele is associated with the hypersensitive GR (Murani et al., 2012), it implies that selection for low post-ACTH cortisol favored the selection of the hypersensitive molecular form of GR. The increased frequency of the hypersensitive GR in the low line was confirmed by genotyping the mutation. The frequency of the mutated allele went from 0.13 in the F0 population to about 0.80 in the G3 low line. It was absent from the G3 high line.

Therefore, lower cortisol levels may result from a more sensitive GR with increased feedback on cortisol production. Consequently, the effects of cortisol on other traits might be limited due to this compensation between cortisol levels and receptor sensitivity. Murani et al. (2016) estimated the effect of the mutation on production traits. For growth traits, including birth weight, they found no significant differences between animals carrying different *NR3C1* alleles. These results contrast with the phenotypic effects of cortisol levels variation associated with genetic polymorphisms of the CBG gene *SERPINA6* on fatness and meat quality traits (Guyonnet-Duperat et al., 2006; Geverink et al., 2006; Ousova et al., 2004).

In the F0 population, the difference between CC and CT individuals was about 25 ng/mL for the cortisol level measured 1 h post-ACTH, and the difference between CT and TT, estimated from animals from the G3 low line only, was about 15 ng/mL. The differences between genotypes for cortisol levels would depend on the conditions of measurements. Murani et al. (2016) observed a difference of 5 ng/mL between CT and TT for cortisol levels measured 1-day post weaning and a difference of 15 ng/mL for cortisol levels measured after slaughter, two conditions considered as stressful. The differences between CC and CT in the same conditions were 18 ng/mL and 11 ng/mL, respectively. As suggested by Murani et al. (2016), the effect of the *NR3C1* c.1829C>T substitution fully appeared after ACTH injection, simulating a stress exposure.

### 3.3 Corticosteroid-binding globulin (CBG) binding capacity

**FIGURE 5:**
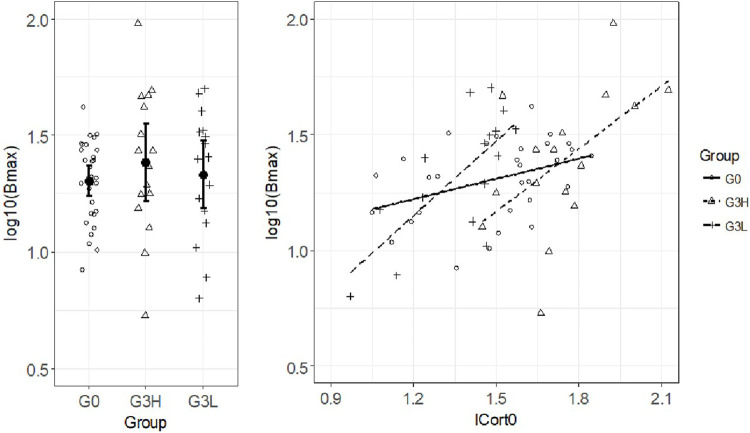
Corticosteroid-binding globulin (CBG) binding capacity. Left panel: CBG binding capacity (Bmax) in the three experimental groups, G0 population and third generation of each selection line with high (G3H) and low (G3L) post-ACTH cortisol levels, log scales. Scatterplot and group means (±SE). Right panel: correlation between CBG binding capacity (log10(Bmax)) and basal cortisol levels, log10 (lCort0).

CBG binding capacity did not differ across experimental groups (P = 0.56; Figure 5), showing that CBG did not contribute significantly to the selection process, contrary to results found in mice selected for plasma corticosterone levels after restraint stress, after 15 to 20 generations of selection (Mattos et al., 2013). In the three experimental groups, CBG binding activity was correlated with basal cortisol levels (G0, r = 0.39, P = 0.03; G3H, r = 0.53, P = 0.03; G3L, r = 0.71, P = 0.002; Figure 5) but not with post-ACTH levels (P = 0.32, 0.93 and 0.058 respectively). Altogether, these results suggest that these two well-demonstrated sources of genetic variation in cortisol levels act independently with possibly different phenotypic consequences.

## 4 Conclusion

The HPA axis has a major influence on both production and robustness traits. It is subject to a large genetic variation and the present experiment in pigs confirms its strong response to selection and the role of the GR gene (*NR3C1*) polymorphism, beside the already demonstrated role of the CBG gene (*SERPINA6*) polymorphism. These and other potential sources of genetic variation will vary according to the polymorphisms of the candidate genes in the population of interest. The consequences of these various sources of genetic variation on production and robustness traits will be further explored in these divergent lines to evaluate their interest for selection of more productive and robust animals.

## Data Availability

The data that support the findings of this study are available on request from the corresponding author.
