# Supplementary figures and images for: Genetic determinism of cortisol levels in pig
Source: Front Genet. 2025 Mar 12;16:1461385. doi: 10.3389/fgene.2025.1461385 (PMC11936974; doi:10.3389/fgene.2025.1461385)

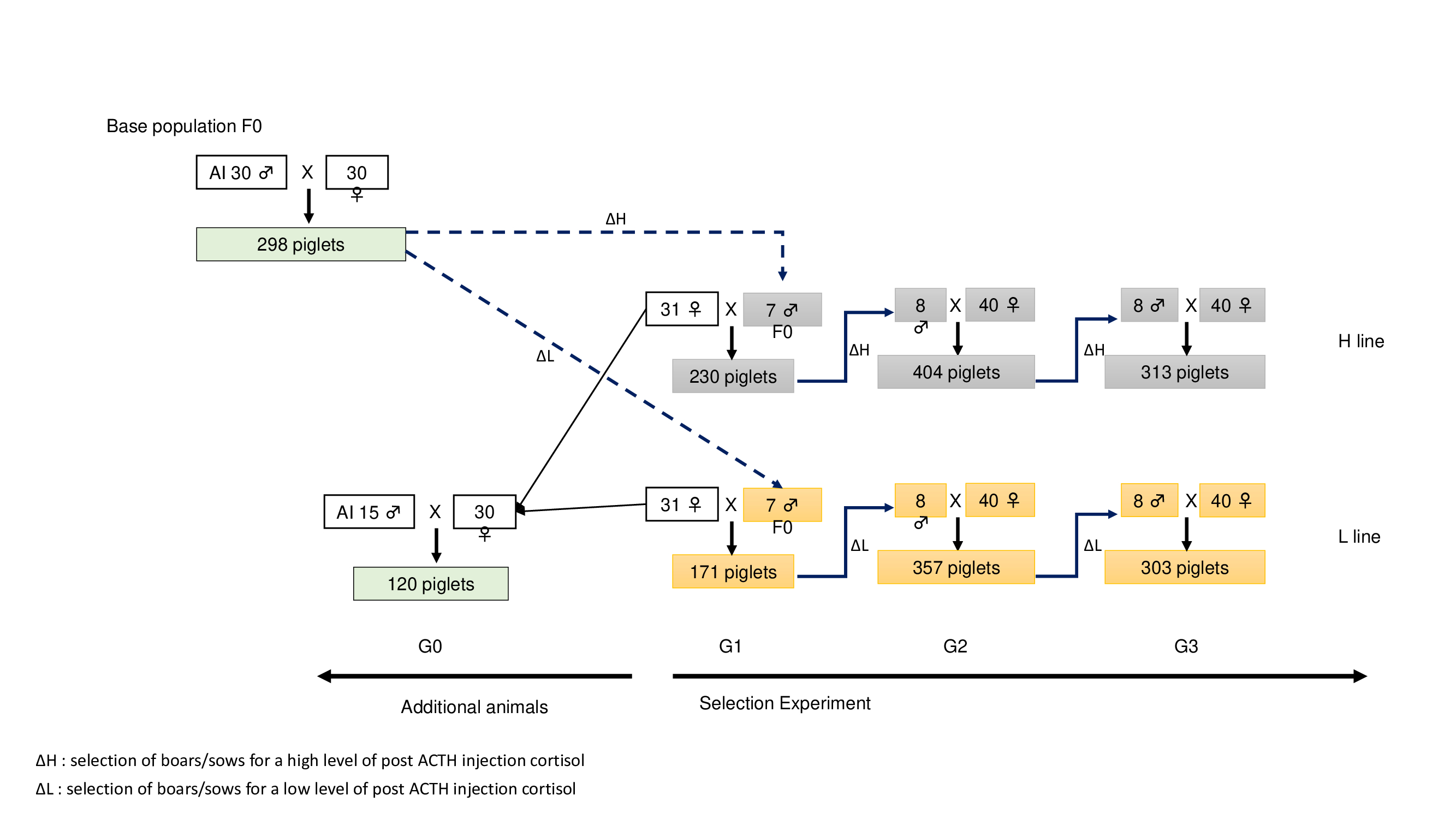

Supplement: Supplementary file 1 [file Image1.jpeg]
